# First Apatite (U-Th)/He and apatite fission-track thermochronology dataset from the Abancay Deflection (Eastern Cordillera, Southern Peru).

**DOI:** 10.1016/j.dib.2021.107748

**Published:** 2021-12-23

**Authors:** Benjamin Gérard, Xavier Robert, Laurence Audin, Cécile Gautheron, Matthias Bernet

**Affiliations:** a GET, Université de Toulouse, CNRS, IRD, UPS, Toulouse, France; b Université Grenoble Alpes, Université Savoie Mont Blanc, CNRS, IRD, IFSTTAR, ISTerre, Grenoble, France; c Université Paris-Saclay, CNRS, GEOPS, Orsay, France

**Keywords:** In-situ apatite, Abancay Deflection magmatic bodies, (U-Th)/He data, Fission-track data, Peru, Eastern Cordillera, Altiplano, Upper crust cooling dynamic

## Abstract

According to their respective temperature sensitivities, Apatite (U-Th)/He (AHe) and apatite fission-track (AFT) thermochronology records the thermal evolution of the upper crust (<5 km) and is a key for distinguishing between different exhumation mechanisms through time-evolving rock uplift, and landscape evolution. We applied these methods to extract the thermal evolution of the upper crust in the Abancay Deflection at the northern edge of the Altiplano (southern Peru). We present 120 single-crystal AHe ages (from 31 samples) and 27 AFT central ages obtained from magmatic bodies across the study area. AHe ages range from 0.6 ± 0.1 to 35.8 ± 2.9 Ma with a satisfactory reproducibility of single-crystal AHe ages with less than 10% averaged dispersion. AFT ages range from 2.6 ± 1.9 to 38.2 ± 4.4 Ma with P(χ^2^) values >5%. This dataset allows exploring the crust evolution from the late-Eocene to the Quaternary. Data processed and interpreted in the related article published in *Tectonics*[6] are stored in PANGAEA repository (108 AHe single-grain ages and 27 AFT ages). We furthermore present in this article 12 extra single-grain AHe ages obtained after the related article publication. We also present the details of fission-track length measurements published in the related article. Thermochronological ages could be reused for testing He diffusion or fission track annealing processes or investigating the broader tectonic/geodynamic evolution of the Andes.

## Specifications Table

**Table:** 

Subject	Geology
Specific subject area	Thermochronological dating methods aimed at determining cooling of rocks.
Type of data	4 Tables – this article 1 Figure – this article 3 Tables (metadata, AHe data, AFT data – from the related research article) stored in the PANGAEA repository [1]
How the data were acquired	Rock samples were collected during a field trip in the Abancay region (Peru) in 2017. For each sample, 5 – 10 kg of rocks were collected from magmatic lithologies. Apatite (U-Th)/He ages were obtained via mass spectrometry [2], [3]. Apatite fission-track ages were obtained using the external detection method (optical microscopy at 1250 x magnification) and calculated with the BINOMFIT program [4].
Data format	Raw Analyzed Filtered
Description of data collection	Apatites were concentrated according to standard crushing, sieving, electro-magnetic and heavy liquid mineral separation techniques. AHe and AFT dating protocols were performed under standard laboratory conditions. (U-Th)/He ages were obtained after He, U, Th, Sm and Ca content measurements for each single-crystal. ^4^He content was determined after single-crystal degassing under vacuum and analyzed by quadrupole mass spectrometry using a calibrated ^3^He spike content. U, Th, Sm and Ca contents were obtained after acid digestion and mass spectrometry analysis with the use of spikes.
Data source / location	• Region: Apurimac and Cuzco • Country: Peru • Latitude and longitude for collected samples/data (coverage): South-bound Latitude: -13.686510°; West-bound Longitude: -73.210850°; North-bound Latitude: -12.647520°; East-bound Longitude: -72.072520°
Data accessibility	With the article (Tables 1–4) and online at PANGAEA [1] Repository name: PANGAEA Data identification number: PDI-26845 Direct URL to data: 10.1594/PANGAEA.929199 - Metadata 10.1594/PANGAEA.929196 - AHe data 10.1594/PANGAEA.929194 - AFT data
Related research article	B. Gérard, X. Robert, L. Audin, P.G. Valla, M. Bernet, C. Gautheron, Differential exhumation of the Eastern Cordillera in the Central Andes: Evidence for south‐verging backthrusting (Abancay Deflection, Peru), Tectonics. (2021). 10.1029/2020TC006314.

## Value of the Data

• The dataset fills a thermochronological data gap in this remote area of Peru.
• These AHe and AFT data allow to explore the tectonic and erosional dynamics of the Eastern Cordillera and northern Altiplano region (Abancay Deflection) [5], [6].
• The data can be integrated at a larger scale and numerically processed for 3D geodynamic investigation (Thermo-kinematics PECUBE forward and inverse modelling [7,8]; Inverse Glide Model [9]).
• The dataset are valuable to unravel the regional thermal evolution and can be further processed by researchers with other geochronological data.
• These thermochronological data allowed us to propose a new view/model of the Abancay Deflection tectonics as a proto-syntaxis rather than a simple fold-and-thrust system [5].
• AFT data can be used for testing or validation of fission-track annealing models [10].

## 1 Data Description

The data files processed in the related research article are available at PANGAEA [1]. All data files can be downloaded in html or Excel format (tables). All variables displayed are clearly specified in the tables. The metadata file contains the sample name, the sampling date, the geological unit sampled, its precise location and elevation information: 10.1594/PANGAEA.929199. The AHe data (10.1594/PANGAEA.929196) and AFT data (10.1594/PANGAEA.929194) tables contain all necessary data and information for their respective analytical protocol.

We also present in this article 12 extra single-crystal AHe ages from 3 samples (AB-17-06, AB-17-15 and AB-17-24). Details regarding the sample locations and lithologies are presented in Table 1. AHe ages are presented in Table 2. Apatite fission-track measurements details (corresponding to the published fission-track ages published in [5]) are given in Table 3. Finally, Table 4 presents the summary of mean fission-track length measurements and the associated errors computed using the HeFTy 1.9.1 program [11].

**Table 1:** Sample locations and bedrock lithologies.

Sample number	Latitude (°S)	Longitude (°W)	Elevation (m)	Lithology	Geologic unit	Pluton period emplacement
AB-17-06	13.07867	72.27952	3696	Granite	Mesapelada pluton	Permian
AB-17-15	12.9652	72.07252	2475	Granite	Colca pluton	Carboniferous
AB-17-24	13.01515	72.96502	3839	Granite	Kiteni pluton	Permian

*Note:* This table is a direct extension of the Table 1 in the related research article [5].

**Table 2:** Apatite (U-Th-Sm)/He data.

Sample number	Morphology	Length (±5 μm)	Width (±5μm)	Thickness (±5μm)	R_s_(μm)	Weight (μg)	F_T_	^4^He (nccSTP/g)	^238^U(ppm)	^232^Th (ppm)	^147^Sm (ppm)	Th/U	eU (ppm)	Age (Ma)	Corrected Age (Ma)	± 1 σ
AB-17-06A	1b + 1 py	125	126	125	76	6.2	0.81	5126	47.9	157.5	90.5	3.3	86	0.5	0.6	0.1
AB-17-06B	2b	120	130	120	82	5.5	0.83	4716	50.1	81.3	44.4	1.6	70	0.6	0.7	0.1
AB-17-06C	2b	124	143	124	86	5.7	0.83	17483	29.4	25.1	90.7	0.9	36	4.1	4.9	0.4
AB-17-06D	2b	103	105	103	70	3.2	0.80	23045	35.2	27.2	95.4	0.8	42	4.6	5.7	0.5
AB-17-06E	1b + 1 py	104	108	104	60	2.5	0.77	20264	40.8	20.8	100.8	0.5	46	3.7	4.8	0.4

AB-17-15A	2b	99	106	139	68	3.2	0.79	10337	4.9	13.4	21.2	2.8	8	10.6	13.4	1.1
AB-17-15E	1b + 1 py	69	97	134	43	1.4	0.68	12410	7.2	15.1	7.5	2.1	11	9.6	14.1	1.1

AB-17-24A	2b	98	99	198	67	4.4	0.79	26281	62.5	91.7	64.7	1.5	85	2.6	3.3	0.3
AB-17-24B	2b	89	102	120	61	2.3	0.77	74866	187.7	101.7	54.7	0.5	212	2.9	3.8	0.3
AB-17-24C	2b	120	133	142	83	4.9	0.83	119789	119.4	20.5	21.7	0.2	124	8.0	9.6	0.8
AB-17-24D	1b + 1 py	91	112	169	57	2.9	0.75	60087	63.3	37.0	17.5	0.6	72	6.9	9.2	0.7
AB-17-24E	1b + 1 py	111	136	145	66	3.4	0.78	62631	59.4	79.0	32.3	1.3	79	6.6	8.5	0.7

*Note:* Morphology refers to the apatite geometry. 2py: 2 hexagonal pyramids; 2b: 2 broken faces; 1b + 1py: 1 broken face & 1 hexagonal pyramid. F_T_ is the alpha ejection correction factor and Rs is the sphere equivalent radius of hexagonal crystal. This table is a direct extension of the Table 2 in the related research article [5].

**Table 3:** Details of fission-track length measurements (AFT dating).

Sample No.	Mineral ID	Track length (μm)	D_par_ (μm)	Angle of the fission track from the c axis (°)
AB-17-05	29	12.42	1.23	0
	31	10.45	1.05	3
	46	12.95	1.47	34
	46	12.17	1.47	2
	53	10.48	1.33	81
	156	10.10	1.31	1

AB-17-06	77	12.71	1.19	83
	107	11.47	1.34	30
	119	12.84	1.33	50
AB-17-07	10	9.56	1.10	45
	96	10.71	1.46	1
	119	9.94	1.11	55
	144	9.65	1.42	80
	179	14.73	1.51	70

AB-17-08	25	10.84	1.26	52
	36	11.11	1.27	58
	42	13.28	1.53	41
	48	11.22	1.19	59
	73	11.22	1.38	83
	89	12.99	1.39	77
	90	13.38	1.43	67
	108	11.23	1.10	83
	108	10.36	1.10	77
	121	10.50	1.40	75
	200	10.36	1.44	47
	215	11.21	1.20	72

AB-17-11	10	9.80	1.32	66

AB-17-13	22	11.25	1.17	60
	22	10.34	1.17	44
	61	10.85	1.11	81
	96	10.86	0.97	90
	175	10.51	0.98	81

AB-17-18	4	11.65	1.78	0
	90	10.34	1.65	80
	160	10.50	1.45	66

AB-17-19	52	10.92	1.42	49
	76	9.73	1.24	44
	105	12.63	1.19	38
	107	12.08	1.22	57
	107	11.79	1.27	52
	122	11.73	1.39	66
	135	10.65	1.23	64
	136	12.71	1.34	40
	165	10.86	1.22	69
	197	11.65	1.22	73

AB-17-22	12	11.62	1.38	82
	21	13.16	1.50	58
	25	9.00	1.50	84
	58	10.03	1.31	43
	58	10.53	1.21	70
AB-17-23	2	11.33	1.27	58
	10	9.65	1.20	69
	12	11.85	1.35	57
	25	11.91	1.20	70
	57	12.24	1.29	81
	57	12.96	1.15	24
	59	12.07	1.24	62

AB-17-25	10	14.22	1.25	63
	10	12.53	1.43	68
	26	12.90	1.53	70
	84	13.27	1.43	62
	107	14.39	1.60	50
	107	13.89	1.76	76
	107	13.96	1.42	63

AB-17-26	12	13.03	1.49	65
	17	10.26	1.23	33
	42	9.92	1.18	67
	180	12.69	1.22	81
	180	11.62	1.34	35
	180	11.22	1.23	68
	180	12.72	1.45	69

AB-17-29	2	12.03	1.72	85
	4	11.33	1.49	4
	4	11.85	1.32	2
	5	11.35	1.32	90
	19	12.15	1.25	43
	19	14.05	1.25	7
	41	10.17	1.5	43
	56	13.01	1.23	86

AB-17-31	8	12.66	1.17	46
	10	12.13	1.23	79
	191	11.67	1.07	82

AB-17-32	40	11.48	1.46	48
	59	11.90	1.18	81

AB-17-33	5	14.98	1.17	57
	25	10.35	1.37	90
	34	12.06	1.28	45

AB-17-36	77	10.62	1.19	40
	80	10.18	1.35	85
	153	11.23	1.34	86

AB-17-37	13	11.31	1.14	68
	13	13.29	1.14	56
	14	11.71	1.10	58

AB-17-38	2	13.55	1.53	68
	2	12.75	1.43	66
	9	12.08	1.75	80
	27	10.75	1.38	73
	27	11.41	1.11	32
	40	11.74	1.58	80
	52	15.03	1.70	76
	53	11.81	1.32	2
	62	12.57	1.52	24
	65	10.95	1.67	60
	67	9.81	1.51	71
	68	10.98	1.64	75
	111	11.54	1.50	86
	150	11.00	1.85	13
	168	13.73	1.57	52
	184	14.11	1.16	71
	195	15.33	2.36	28
	195	14.60	1.84	65
AB-17-39	3	11.32	1.25	52
	10	11.34	1.73	2
	20	10.14	1.87	54
	45	12.35	1.43	74
	96	11.73	1.85	53

AB-17-40	11	9.03	1.57	43
	45	10.45	1.30	72
	174	10.88	1.53	61
	167	11.11	1.18	40
	198	9.73	1.22	56
	205	13.00	1.30	68
	209	12.41	1.31	70

AB-17-42	49	10.82	1.64	90
	131	11.55	1.66	2
	196	10.91	1.51	57
	197	12.89	1.73	69
	197	11.48	1.78	75
	198	11.68	1.44	64
	209	12.71	1.37	2

AB-17-44	3	12.13	1.80	53
	7	12.22	2.00	68
	14	12.96	1.62	68
	31	9.01	1.48	72
	40	10.23	1.66	57
	94	9.25	1.47	72
	95	10.11	1.83	86
	111	12.40	1.71	79
	112	14.21	1.96	45
	136	11.93	1.77	67
	136	11.26	1.81	83
	136	14.57	2.03	79
	136	10.87	1.82	85
	144	9.35	1.85	3
	155	8.68	1.96	85
	164	9.54	1.79	28
	164	9.44	2.01	71
	170	8.57	1.82	55
	171	16.61	1.95	67
	171	10.75	1.82	52
	216	14.61	1.55	59

AB-17-55	12	11.26	0.90	4
	48	10.81	0.87	87
	64	9.66	0.99	81
	69	10.62	1.19	5
	69	10.83	1.02	2
	100	11.08	0.84	53

Notes: Horizontal track lengths were measured dry at 1,250X magnification under an Olympus BX51 optical microscope, using the FTStage 4.04. D_par_: fission -track etch figure diameter. c axis: elongation axis of the apatite. Error of individual track lengths measurements is on the order of 8%.

**Table 4:** Summary of mean fission-track length measurements (AFT dating).

Sample No.	n	Mean track length(μm)	+/- 1 sigma error	c-axis projected mean track lengths(μm)	+/- 1 sigma error
AB-17-05	6	11.43	1.22	12.00	1.44
AB-17-06	3	12.34	0.76	13.67	1.05
AB-17-07	5	10.92	2.18	12.61	1.84
AB-17-08	12	11.47	1.10	13.45	0.72
AB-17-11	1	9.80	0.00	12.46	0.00
AB-17-13	5	10.76	0.35	13.03	0.47
AB-17-18	3	10.83	0.71	12.50	0.74
AB-17-19	10	11.47	0.93	13.26	0.62
AB-17-22	5	10.87	1.59	13.15	0.98
AB-17-23	7	11.72	1.03	13.51	0.55
AB-17-25	7	13.59	0.70	14.84	0.41
AB-17-26	7	11.64	1.24	13.38	1.08
AB-17-29	8	11.99	1.16	13.14	1.21
AB-17-31	3	12.15	0.50	13.91	0.14
AB-17-32	2	11.69	0.30	13.52	0.58
AB-17-33	3	12.46	2.34	14.04	1.43
AB-17-36	3	10.68	0.53	12.90	0.64
AB-17-37	3	12.10	1.05	13.83	0.61
AB-17-38	18	12.43	1.62	13.81	1.32
AB-17-39	5	11.38	0.81	12.89	1.07
AB-17-40	7	10.94	1.40	12.97	1.10
AB-17-42	7	11.72	0.81	13.18	0.90
AB-17-44	21	11.37	2.25	13.33	1.66
AB-17-55	5	10.71	0.56	11.97	1.67

Note: Mean track length and c-axis projected mean track lengths and their error estimates were calculated using the HeFTy 1.9.1. program of [11]. n: number of track lengths measured.

## 2 Experimental Design, Materials and Methods

The Andes are the classical and systemic example of a Cordilleran-type active orogen, with mountain-building related to a subduction zone. Despite numerous studies of the exhumation setting, several phases of its long-term orogeny remain unsolved spatially or poorly documented. To address this, new thermochronological data are provided in this study. Apatite (U-Th)/He (AHe) and apatite fission-track (AFT) thermochronology are based on He and fission track production during alpha decay of ^238^U, ^235^U, ^232^Th, and ^147^Sm and fission decay of ^238^U, respectively. As a result, associated ^4^He and fission tracks will accumulate within apatite crystals. Because of its temperature sensitivity, apatite low-temperature thermochronology records the thermal evolution of the upper crust (<5 km) and is a key for distinguishing between different exhumation mechanisms through time-evolving rock uplift, and landscape evolution. We applied these methods to extract the thermal evolution of the upper crust in the Abancay Deflection at the northern edge of the Altiplano (southern Peru). Thermochronological data presented in this study were derived from the analysis of magmatic apatites. We collected samples of igneous rocks (Granite, Monzonite, Diorite, Gabbro, Orthogneiss) across the Abancay Deflection in order to have optimal thermochronological data coverage with the aim of carrying out thermo-kinematic numerical modelling of the upper crust in this uninvestigated remote part of the Peruvian Andes. The Abancay Deflection is located at the northern edge of the Peruvian Altiplano. Its hinge-like morphology is emphasized by the deflection of its fault patterns, its internal cordilleras and its captured hydrographic networks from the overall elongation axis of the Andes (deflection > 45°). The study area encompasses two distinct morphotectonic regions; the Altiplano southward, characterized by Eocene plutons emplaced into Meso-Cenozoic sediments, and the Eastern Cordillera northward, characterized by Permo-Triassic plutons emplaced into Paleozoic metasediments [5]. These plutons were preferentially targeted for sampling during the fieldwork.

The experimental design, materials and methods description hereafter is based on the method section published in [5]. Parts of the text may be similar. The paragraphs presented hereafter are, however, more detailed and information about the analytical protocol and procedure have been added.

As far as possible, we sampled *in-situ* outcropping bedrock in the field (5-10 kg per sample). Apatites were concentrated according to standard crushing, sieving, electro-magnetic and heavy liquid mineral separation techniques. Magmatic rock samples were crushed cold and sieved at the Géode Laboratory (Lyon, France) to extract the 100-160-μm grain size fractions. The mineral separation techniques were performed at the GeoThermoChronology (GTC) platform within the ISTerre Laboratory (Université Grenoble Alpes, France).

**Fig. 1:**
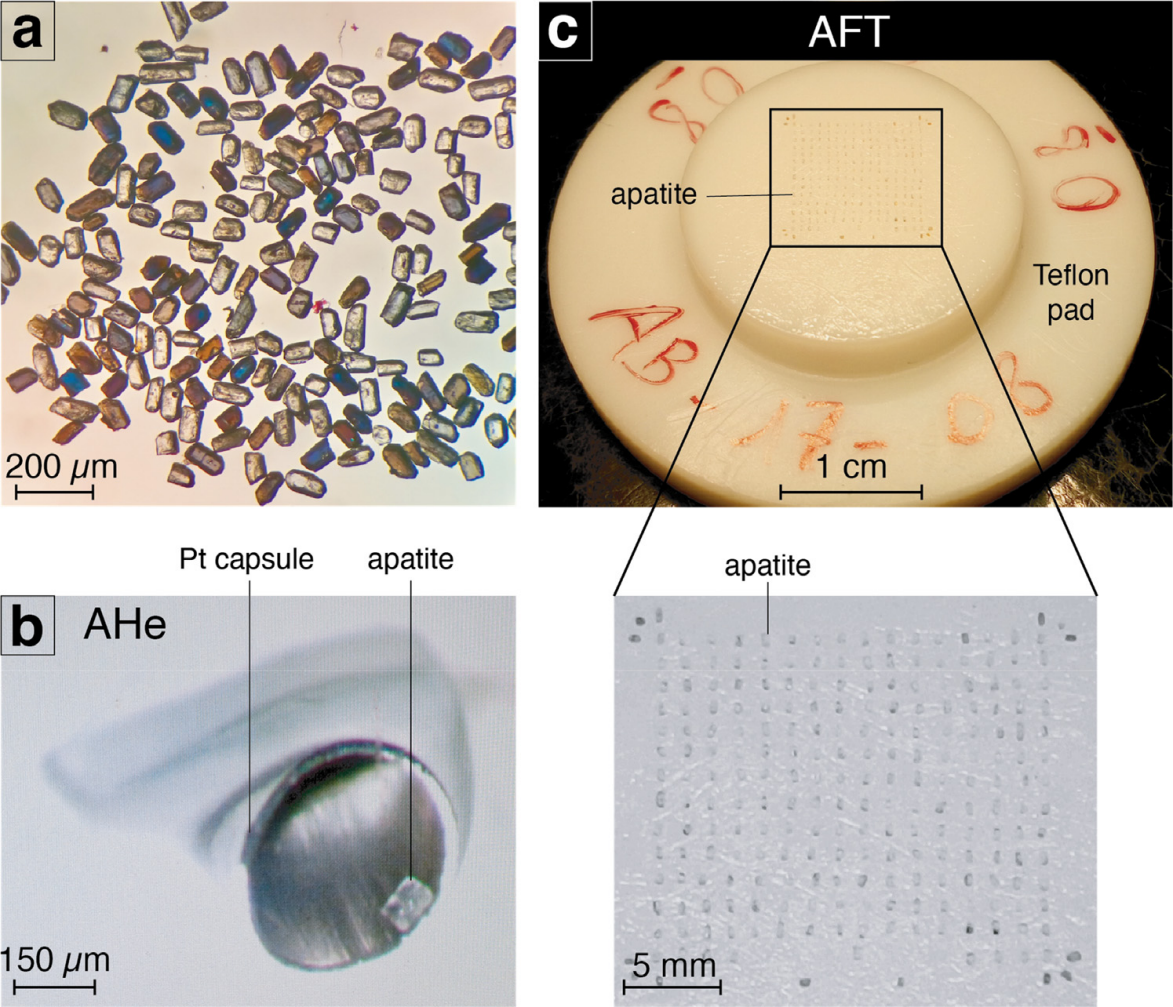
Sample preparation protocols for AHe and AFT dating. (a) Apatites during manual selection under binocular microscope. (b) Apatite encapsulation into a platinum cylinder for AHe dating protocol after initial sorting and selection. (c) Apatites arranged on a Teflon pad before casting the epoxy resin (example from the sample AB-17-08).

In the context of AHe dating, apatite crystals were carefully selected a under binocular microscope to manually select minerals without fractures and/or inclusions. This strict selection was performed in order to prevent biases in the computation of AHe ages. We removed minerals that included these defects from the analytical procedure to avoid any potential additional source of ^4^He, or He diffusion artifacts [12] (Fig. 1a). Selected minerals were then measured in three dimensions (length, width and thickness) with a digital ruler. We determined the individual grain geometry (to compute the sphere equivalent radius of hexagonal crystal (R_s_)) and calculated the α-ejection correction factor using the Qt_FT program [13], [14], [15] (based on Monte Carlo simulation). The α-ejection correction factor was computed from the crystal dimensions. This takes into account the loss of He atoms located beneath the grain boundaries. Small crystals would consequently lose a larger proportion of He during ejection than large grains. We selected five replicates per sample for analysis. Some samples present less than five aliquots analysed because the number of suitable apatites for AHe dating was too low or because He content extracted from the mineral after analysis was too close from the blank to be reliable. After this first phase of identification, we encapsulated individual apatites in platinum tubes (Fig. 1b). Each platinum tube and the apatite it contains were heated under high vacuum conditions at high temperature (1,050 ± 50°C) twice for 5 min at GEOPS Laboratory (Université Paris-Saclay, France). The released ^4^He gas was mixed with a known amount of ^3^He, purified, and the gas was analysed using a Prisma Quadrupole. The ^4^He content was determined by isotope dilution method. Subsequently, apatite crystals were dissolved in 50 μL of HNO_3_ 5 N solution containing known amount of ^235^U, ^230^Th, ^149^Sm, and ^42^Ca. The solution was heated at 70°C for 3 h and 900 μL of distilled water was added. The final solution was analyzed using an ELEMENT XR ICP-MS (CCT-QMS series II at the LSCE laboratory at Gif sur Yvette, France) and the ^238^U, ^230^Th, and ^147^Sm concentrations and apatite weights (using the Ca content [16]) were obtained. Finally, AHe ages were computed following the methodology detailed in [3]. Durango apatite crystals were also analyzed over the same period to ensure the data quality. The 1σ error on each AHe age amounts to 8%, reflecting the analytical error and the uncertainty on the ejection factor correction [3].

Regarding the AFT dating, initial mounting and fission tracks revealing procedures were done at the GTC Laboratory (ISTerre, Grenoble, France). Almost 3200 apatites were mounted in epoxy resin in Teflon molds (Fig. 1c), polished, and etched for 20 s at 21°C using a 5.5 M HNO_3_ solution to reveal spontaneous fission tracks. Using the external detector method [17], [18], [19], all samples were irradiated together with Durango and Fish Canyon Tuff age standards and IRMM540R dosimeter glasses at the FRM II reactor (Munich, Germany). Tracks were counted and horizontally confined track lengths were measured dry at 1,250X magnification under an Olympus BX51 optical microscope, using the FTStage 4.04 program at ISTerre. Only TinT (Track in Track) were measured following recommendations from [20]. We performed AFT central ages computation using the BINOMFIT program [4] according to the ζ-calibration technique [17,19]. Measurement of five Durango and five Fish Canyon Tuff age standards allowed establishing a ζ-value of 275 ± 12 to be constrained for the operator B. Gérard.

These thermochronological data were subsequently processed in thermo-kinematics model (PECUBE; [8]) after consideration regarding the geothermal gradient in order to test the upper crust evolution of the Abancay Deflection area. Indeed, PECUBE modelling allows quantification of thermal histories for rock particles at depth in exhumation or burial contexts. Details regarding thermal assumptions and cooling/exhumation histories for the crust are detailed and discussed in [5].

## Ethics Statements

Nothing to declare.

## CRediT authorship contribution statement

**Benjamin Gérard:** Conceptualization, Investigation, Data curation, Writing – original draft, Visualization. **Xavier Robert:** Conceptualization, Investigation, Writing – review & editing, Supervision, Funding acquisition. **Laurence Audin:** Conceptualization, Investigation, Writing – review & editing, Supervision, Funding acquisition. **Cécile Gautheron:** Methodology, Validation, Resources, Data curation, Writing – review & editing, Funding acquisition. **Matthias Bernet:** Methodology, Validation, Resources, Data curation, Writing – review & editing.

## Declaration of Competing Interest

The authors declare that they have no known competing financial interests or personal relationships that could have appeared to influence the work reported in this paper.
